# Developing a roadmap to improve trial delivery for under-served groups: results from a UK multi-stakeholder process

**DOI:** 10.1186/s13063-020-04613-7

**Published:** 2020-08-01

**Authors:** Miles D. Witham, Eleanor Anderson, Camille Carroll, Paul M. Dark, Kim Down, Alistair S. Hall, Joanna Knee, Rebecca H. Maier, Gail A. Mountain, Gary Nestor, Laurie Oliva, Sarah R. Prowse, Amanda Tortice, James Wason, Lynn Rochester, On behalf of the INCLUDE writing group

**Affiliations:** 1 NIHR Clinical Research Network Cluster E, Campus for Ageing and Vitality, Newcastle University, Newcastle, NE4 5PL UK; 2 NIHR Newcastle Biomedical Research Centre, Newcastle University and Newcastle upon Tyne Hospitals NHS Trust, Newcastle, UK; 3 University of Plymouth, Faculty of Health, Plymouth, Devon UK; 4 NIHR Manchester Biomedical Research Centre, University of Manchester and Northern Care Alliance NHS Group, Manchester, UK; 5 Cardiology Department, Leeds General Infirmary, Leeds, LS1 3EX UK; 6 NIHR Clinical Research Network Coordinating Centre, 21 Queen Street, Leeds, LS1 2TW UK; 7 Newcastle Clinical Trials Unit, 1-4 Claremont Terrace, Newcastle University, Newcastle upon Tyne, NE2 4AE UK; 8 Centre for Applied Dementia Studies, University of Bradford, Bradford, UK; 9 NIHR Clinical Research Network Coordinating Centre, London, UK; 10 Institute of Applied Health Sciences, University of Aberdeen, Aberdeen, UK; 11 NIHR Yorkshire and Humber Local Clinical Research Network, Yorkshire and Humber, UK; 12 Population Health Sciences Institute, Newcastle University, Newcastle upon Tyne, UK; 13 MRC Biostatistics Unit, University of Cambridge, Cambridge, UK; 14 Translational Clinical Research Institute, Newcastle University, Newcastle upon Tyne, UK

**Keywords:** Trials, Roadmap, Under-served, Recruitment, Stakeholder

## Abstract

**Background:**

Participants in clinical research studies often do not reflect the populations for which healthcare interventions are needed or will be used. Enhancing representation of under-served groups in clinical research is important to ensure that research findings are widely applicable. We describe a multicomponent workstream project to improve representation of under-served groups in clinical trials.

**Methods:**

The project comprised three main strands: (1) a targeted scoping review of literature to identify previous work characterising under-served groups and barriers to inclusion, (2) surveys of professional stakeholders and participant representative groups involved in research delivery to refine these initial findings and identify examples of innovation and good practice and (3) a series of workshops bringing together key stakeholders from funding, design, delivery and participant groups to reach consensus on definitions, barriers and a strategic roadmap for future work. The work was commissioned by the UK National Institute for Health Research Clinical Research Network. Output from these strands was integrated by a steering committee to generate a series of goals, workstream plans and a strategic roadmap for future development work in this area.

**Results:**

‘Under-served groups’ was identified and agreed by the stakeholder group as the preferred term. Three-quarters of stakeholders felt that a clear definition of under-served groups did not currently exist; definition was challenging and context-specific, but exemplar groups (e.g. those with language barriers or mental illness) were identified as under-served. Barriers to successful inclusion of under-served groups could be clustered into communication between research teams and participant groups; how trials are designed and delivered, differing agendas of research teams and participant groups; and lack of trust in the research process. Four key goals for future work were identified: building long-term relationships with under-served groups, developing training resources to improve design and delivery of trials for under-served groups, developing infrastructure and systems to support this work and working with funders, regulators and other stakeholders to remove barriers to inclusion.

**Conclusions:**

The work of the INCLUDE group over the next 12 months will build on these findings by generating resources customised for different under-served groups to improve the representativeness of trial populations.

## Introduction

Evidence from randomised controlled trials constitutes the most rigorous test of efficacy, effectiveness and safety for healthcare interventions. Such evidence is essential to identify both what works and what does not work, and underpins the highest grade of recommendations in guidelines that shape clinical practice [1]. However, populations recruited into trials are often not representative of the target population who require evidence about interventions. For example, participants in heart failure trials are on average 20 years younger than those seen in clinical practice; patients in cancer trials lack the comorbidity commonly seen in clinical practice; and patients in diabetes trials are not the ethnically diverse populations typically seen in clinical practice [2–4]. The consequence of this disconnect between trial populations and the real world are manifold—treatment benefits and harms seen in trials may not translate to those seen in clinical practice [5], and important findings specific to different populations may be missed. Trial interventions may not be deliverable to, or may not benefit, all groups within a population and only by including a range of groups can researchers, patients, clinicians and policymakers know that results are applicable to all within a population. Whilst including under-served groups may require more time, money and effort, the value of the results is likely to be higher. Finally, this lack of representation could be interpreted as discriminatory against some groups.

Some reasons for this disconnect between research and practice have been previously described. Trials often seek a narrowly defined, homogenous population to reduce variance and hence sample size, and the imposition of stringent inclusion and exclusion criteria leads to unrepresentative trial populations [2, 6, 7]. Recruitment strategies and trial logistics may also act as barriers to entry and retention. Examples include recruiting via tertiary care centres, or in urban environments far from rural populations, failing to provide taxi transportation for people who have poor mobility, or failing to provide translated study information for those who cannot read a given language [8]. Pressure from funders to recruit rapidly may also favour approaching groups who are easy to recruit, rather than recruiting a more representative, but harder to recruit, range of participants.

At present, a systematic approach to understanding and overcoming the lack of representativeness of trial populations has not been attempted. In the UK, the National Institute for Health Research (NIHR), a major funder of clinical trials designed to underpin guidelines and clinical practice, identified the need for a workstream to address this issue, latterly named the ‘Innovations in Clinical Trial Design and Delivery for the Under-served’ (INCLUDE) project. This paper reports the results of the first phase of this work which had the following key objectives: to create a framework to allow the identification of groups who are historically under-represented in trials, to identify barriers and drivers to inclusion and to facilitate innovations in trial design and delivery to improve inclusion of under-represented groups.

## Methods

**Fig. 1:**
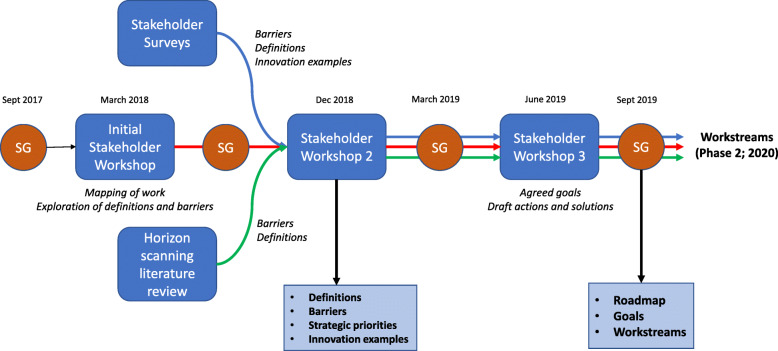
Components of the programme. SG steering group

A series of tasks and events together addressed the objectives of the programme, which we describe below (summarised in Fig. 1).

### Steering group and stakeholder group meetings

Three stakeholder group meetings were held—in March 2018 as a project initiation workshop, December 2018 as a prioritisation and definition consensus meeting and June 2019 as a goal and future work consensus meeting. Stakeholder groups relevant to the process were mapped by the project steering group; representatives were selected through organisational structures and by recommendation from within each stakeholder organisation. Stakeholders invited to these meetings included NIHR national speciality leads and members from the NIHR Clinical Research Network, public and patient groups and charities, practitioners and healthcare professionals, the life sciences industry, funders and regulatory bodies. Additional meetings of a smaller steering group, comprising NIHR project and research network staff, clinical academics, trialists and leaders of public engagement activities, were held between these meetings to interpret and refine findings, provide strategic direction to the project, and to plan the content of the stakeholder group meetings.

### Literature review

A targeted scoping review of published literature was undertaken by the NIHR Innovation Observatory relating to the concept of under-representation as it appears within clinical trial design and/or delivery. Keywords identified by the study team included under-represented groups, hard to reach groups, outside of the standard medical settings, barriers to participation and patient participation. A combination keyword method for the identification and inclusion of relevant studies was employed, including searches undertaken in MEDLINE, Embase, Cochrane Database of Systematic Reviews, Database of Promoting Health Effectiveness Reviews (DoPHER), Trial Register of Promoting Health Interventions (TRoPHI), Scopus, Cumulative Index to Nursing and Allied Health Literature (CINAHL) and PsycINFO. Searches were conducted in April and May 2018 and were limited to a 5-year timeframe, starting 1 March 2013. Results not in English were excluded, as were results describing under-representation of an intervention as opposed to a group of participants. Letters, commentaries, editorials and animal studies were also excluded. A sample search strategy is shown in Additional file 1. Retrieved references were deduplicated in EndNote before scrutiny by the research team for relevance. All titles were scrutinised; clearly irrelevant titles were removed, and the remaining candidate studies proceeded to abstract review, where irrelevant abstracts were further removed. Much of the available literature focussed on patients with cancer; thus, studies were divided into those focussing on cancer and non-cancer populations. Key findings were extracted by a research team member (SP) before further grouping into themes by the steering group. Final results were then fed into the work of the stakeholder meetings.

### Surveys

Two surveys were designed, asking very similar questions to two distinct target groups. The first group constituted stakeholders identified from potentially under-represented groups. Stakeholders were mapped by the steering group with additional suggestions from the wider stakeholder group. The survey was sent to charity users, public and patient group representatives, NIHR Clinical Research Network research ambassadors and Newcastle University VOICE (Valuing Our Intellectual Capital and Experience, a public engagement organisation) members. The second group constituted professional stakeholders, drawn from a range of clinical research leaders within the NIHR Clinical Research Networks, charities, clinical trial units, other parts of the NIHR organisation, industry, NHS and universities. As with the professional group, recipients were identified by the steering group and wider stakeholder group, with organisations suggesting additional contacts within their organisations. Surveys were hosted on SurveyMonkey and links sent via targeted emails. Results from the surveys were analysed by thematic analysis of free-text responses. The survey questionnaires are presented in Additional file 2.

## Results

### Initial stakeholder meeting

At the initial stakeholder meeting, held in March 2018, 17/23 (74%) of participants answered ‘no’ to the statement: “Do you feel that there is a sufficiently consistent definition of what is meant by ‘under-represented groups’ in clinical trials across stakeholders?”. No stakeholder at the meeting agreed with the statement, and 6/23 (26%) held no opinion. Stakeholders provided examples of under-represented groups (for example older people, pregnant women, people from traveller communities, people from Black, Asian and Minority Ethnic [BAME] communities), innovative practice aimed at successfully engaging with and recruiting such groups (for example using researchers from within the communities, long-term engagement and co-design of studies by communities) and suggested that a single definition may not capture all groups likely to be under-represented.

### Targeted scoping review of definitions for under-served groups

A total of 58 full-text papers were eligible for review; a description of all considered full-text papers is given in Additional file 3. The majority (39/58) of papers studied patients from the USA; 15 studies reported data from globally distributed sources, often as part of systematic or narrative reviews, and 4 studies reported data collected from other countries. Types of under-represented groups were similar in both cancer and non-cancer literature, including broad groupings such as children, older people, patients with multimorbidity, pregnant women, ethnic minorities, the socioeconomically disadvantaged, people outside usual primary and secondary healthcare settings and those with cognitive impairment. The majority of the literature (31/58 studies) engaged with a specific subgroup or population. Common thematic barriers identified in the literature that may lead to under-representation include the presence of comorbidities, logistical barriers (including lack of transport, remoteness of study sites from usual abode), information not available in a language or form to allow easy comprehension, low trust or low engagement with healthcare services and low awareness of clinical trial opportunities. Reviews retrieved as part of the literature review reinforced that under-representation of some groups (especially women and ethnic minority groups) was still prevalent in major disease areas such as cancer and cardiovascular disease trials, and also highlighted that many studies do not even collect or report information to enable a judgement on whether a group was under-represented in a particular trial [9, 10].

### Surveys

Seventy people completed the participant stakeholder on-line survey. Respondents were not asked directly for details regarding which under-served group they were a member of but reported the route by which they had received the invitation to participate. Participants received the invitation via VOICE in 11 (16%) of cases, via their role as Patient Research Ambassadors in 8 (11%) of cases, from other sources within the NIHR Clinical Research Network in 11 (16%) cases, via medical charities in 6 (9%) of cases and from other sources in 19 (27%) of cases. The remaining 15 (21%) did not respond to this question. Forty-one respondents (59%) agreed that it was difficult to be clear about what under-representation means.

One hundred and one professionals completed the survey. Thirteen (13%) were research nurses, and 13 (13%) were academic researchers. Fourteen (14%) were staff from clinical trial units, including unit directors. Twelve (12%) were research managers, nine (9%) were CRN Specialty leads and a further 11 (11%) held other CRN roles. Smaller numbers of contributors represented industry (6 [6%]), the NHS (6 [6%]), PPI engagement professionals (5 [5%]) and charities (2 [2%]), with 10 (10%) not responding to this question. Fifty-six respondents agreed that a universal definition of under-representation could not be given, 14 disagreed with the statement and 17 stated that they did not know.

Table 1 shows the under-served groups that were identified by the stakeholder, professional and patient representatives. A small number of respondents also suggested groups that they judged not to be under-served: these were children, older people, people living with frailty, people with multiple conditions, women and socioeconomically disadvantaged groups. These groups overlap with groups judged by others to be under-served, but no respondent named a group as both under-served and not under-served.

**Table 1:** Under-served groups identified from stakeholder groups and surveys

**Groups by demographic factors**	
Age extremes (e.g. under 18 and over 75)	
Women of childbearing age	
Black, Asian and Ethnic Minorities (BAME)	
Male or female sex (depending on trial context)	
LGBTQ/sexual orientation	
Educational disadvantage	
**Groups by social and economic factors**	
In full-time employment	
Unemployed/low income	
Military veterans	
People in alternative residential circumstances^a^	
People living in remote areas	
Religious minorities	
Carers	
People not fluent in the majority language	
People who do not attend regular medical appointments	
People in multiple excluded categories	
Socially marginalised people	
Stigmatised populations	
Looked after children	
**Groups by health status**	
Mental health conditions	
People who lack the capacity to consent for themselves	
Cognitive impairment	
Learning disability	
People with addictions	
Pregnant women	
People with multiple health conditions	
Physical disabilities	
Visually/hearing impaired	
Too severely ill	
Smokers	
People living with obesity	
**Groups by disease-specific factors**	
Rare diseases and genetic disease subtypes	
People in cancer trials with brain metastases	

^a^This category includes migrants, asylum seekers, prison populations, care homes, traveller communities and homeless

Twenty respondents went on to provide their own definition of under-representation. These definitions were broadly grouped into three areas: (1) inclusion, (2) epidemiology and (3) miscellaneous. Inclusion includes all submissions that considered under-representation to be defined by poor access to trials in certain population groups. Epidemiology is those that consider under-representation to be defined in epidemiological terms, i.e. the trial participant population demographics failing to mirror the demographics of the target population in the ‘real world’. Miscellaneous includes those submissions that could not be grouped (*n* = 7). Some of these submissions did attempt to provide definitions of under-representation, for example ‘People who do not usually feature in the demographics of those recruited to clinical trials’ (participant ID 10210064831). However, many were commentary on the issue rather than definition, for example ‘I have found - for example - none of the studies I have been involved with have been gender specific, however, nearly always I screen and recruit more males than females…’ (participant ID 10210238642). Table 2 summarises the barriers to trial inclusion identified by the participant and professional surveys.

**Table 2:** Barriers to inclusion

Barrier	
Barriers relating to physical disability	
Difficulties in consenting for another person	
Feeling unqualified to take part (e.g. due to lack of education)	
Lack of available trials/poor trial promotion	
Lack of effective incentives for participation	
Lack of interest in research	
Lack of trust in trials	
Negative attitudes to the concept of research	
Negative financial impact	
Potential participants refusing to accept their health condition	
Poor consent procedures	
Requirement for additional carer time to aid participant	
Participant risk perception	
Specific cultural barriers	
Specific health fears (e.g. hospitals, needles)	
Treatment centres not set up for research	
Trials asking too much for participation	
Unwilling to receive placebo	

### Output from second stakeholder meeting

The consensus meetings considered the information from the literature review and surveys, adding to and refining the list of examples of innovative practice that had emerged from the survey. A consensus emerged that ‘under-served groups’ be used as the preferred term for describing the target for the group as this was felt to avoid any attachment of blame for low levels of participation. It also ensured that the onus was firmly on the research infrastructure and process to find ways to provide an appropriate solution or service to prospective research participants. Consensus was also reached that a single universal definition for what constitutes an under-served group was unlikely to be possible. There was consensus that the definition is context-specific, but that important elements include a mismatch between a population affected by a condition and those enrolled in studies; a lack of opportunity to participate due to study design, logistical or cultural barriers; and a lack of suitability of the intervention under study for a particular group due to physiological, logistical or cultural factors. Stakeholders listed their priorities for the work of the group over the coming year to achieve the vision of ‘better healthcare through more inclusive research’; the top six priorities (in priority order) were:

1. Embed research within healthcare to improve access
2. Need for a set of information resources and training to support research teams, community representatives and clinical/support staff/other key stakeholders (including funders) in building capacity and sustainable engagement to enable development and delivery of research to the under-served
3. Funders to engage with and enable provision for under-served groups, making trials appropriate to the real-world group(s) targeted by the intervention
4. More patient-centredness throughout the research process (for example information and consent, dissemination); Community Partnered Participatory Research (CPPR)
5. Nationwide publicity drive on what clinical trials are and why they matter—TV series, features, etc.
6. Baseline measurement to assess the current situation and to help document improvement

Following the second consensus meeting, the steering group reviewed all the project outputs and proposed a set of overarching goals together with an initial list of potential solutions and actions to achieve these goals. The project was branded as ‘Innovations in Clinical Trial Design and Delivery for the under-served’ (INCLUDE) at this point.

### Output from the third stakeholder meeting

At the final meeting, the set of goals identified from the work to date was presented to the stakeholder group. The group generated further potential solutions and high-level actions, and the steering group then generated four key objectives of equal importance for future work:

A) Develop Community Partnered Participatory Research (CPPR) building long-term relationships with under-served groups and opportunities for participation
B) Develop training resources to design and deliver trials for under-served for all stakeholders involved, and tailor resources to the needs of specific groups
C) Develop infrastructure and systems to reach, engage, recruit and retain under-served groups
D) Work with funders, regulators and other stakeholders to ensure barriers in funding, regulations and policies are removed to enable inclusion of under-served groups

**Fig. 2:**
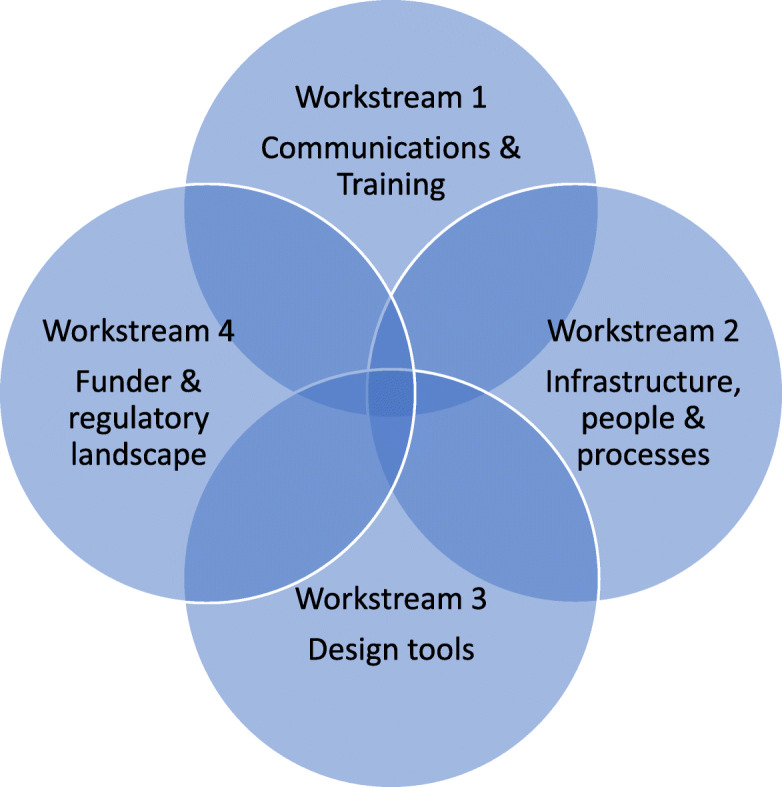
Workstreams to take forward in the programme

To address these objectives, the steering group identified four overlapping workstreams to take forward; each workstream addresses multiple objectives. These are shown in Fig. 2.

### Final strategic roadmap

**Fig. 3:**
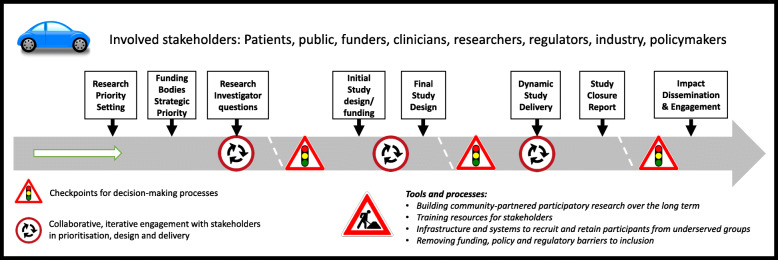
Roadmap to guide initiatives to improve inclusion of underserved groups into clinical research. Processes are embedded in the context of ethics and regulatory requirements and evolving digital technology developments. Boxes represent key points for considering inclusion of underserved groups over the lifecourse of the study

The steering group combined all the outputs from the project surveys, scoping review and stakeholder meetings to produce a strategic roadmap for initiatives to improve access to clinical research for under-served groups. The roadmap is intended to guide groups wishing to focus on the needs of specific under-served groups, and to help researchers work with under-represented groups; it does not aim to provide solutions (which will be specific to each under-served group) but provides a structure for both future research and for initiatives to address barriers to participation that have been identified during the project. The final roadmap is shown in Fig. 3.

## Discussion and future directions

The INCLUDE project has identified a preferred label (‘under-served groups’) to support conversations around improving the inclusion of under-served groups in clinical research, has identified a range of example under-served groups, has identified common barriers to participation and has produced a strategic roadmap and plan to inform future work in this field. The recognition that clinical research often fails to engage with groups that it needs to is not new, and several papers included in our literature review reinforce that key groups such as older people, ethnic minorities and women are still under-represented in clinical studies. Worse still, data are lacking in many study reports on key study characteristics that are needed to understand if under-served groups are included in clinical research. Despite the recognition that a problem exists, attempts to address the problem in a systemic way have been lacking to date. Most studies on barriers to recruitment originate from the USA, and many are specific to cancer research [11, 12]. Examples exist of research exploring barriers and solutions to participation in other areas, for instance older people, those from ethnic minorities and those in acute or critical care [13–17]. Different areas of research have different barriers in engagement to overcome; hence, a tailored approach is required. Nevertheless, there are likely to be common, generic issues (such as funding and regulatory disincentives) that could be tackled by generic action, and a framework for future action such as we propose here is also likely to reduce duplication of effort, redundancy and hence research waste in identifying good practice and approaches to the problem.

The barriers identified by our surveys accorded well with those identified in our literature search and encompassed those initially suggested by our stakeholder group. Previous work has identified themes of trial awareness, opportunity, acceptance of enrolment, perceptions of the research process, logistical barriers and whether trial processes were able to fit into participants’ existing lives [18, 19]. These issues group in a similar way to the barriers we identified. Although we did not find initiatives with exactly the same aim and scope as our work, there are others that either overlap or are complementary. Of particular note are initiatives to improve recruitment to and retention in trials [20] and ways to improve trial design methods [21]. Our work is well placed to complement and enhance these and other initiatives.

### Strengths

The work we describe was successful in obtaining consensus on strategic guidance for future work in this area and had the strength of involving a wide range of stakeholders including those from under-served groups. The use of a range of methods, drawing on expert opinion, published literature and focussed surveys, provided the ability to triangulate data from multiple sources, strengthening the final set of objectives and the final roadmap. Although much of the existing literature surveyed was conducted in the context of clinical trials, the work of the INCLUDE project stakeholder and steering group kept a perspective that encompassed all types of clinical research, and the outputs from the project should therefore be of relevance to a broad range of clinical research, not just trials.

### Limitations

The work we describe was conducted as a focussed, time-limited project. It was not conceived or executed as a pure scientific investigation, and thus, some compromises were necessary in terms of depth and extent of enquiry. We did not attempt to apply a full systematic review methodology to our literature review, but instead used a focussed scoping review method which better fitted the needs of the project. Although we were successful in obtaining a wide range of stakeholder views, we acknowledge that in terms of numbers, representation was stronger from the academic and research delivery communities than from other areas such as commercial or charity representatives. Not all barriers identified by respondents and participants will apply to all groups or all trials; barriers are context-dependent, and not all barriers will be surmountable for a particular trial. A better understanding of what respondents meant for some barriers is needed; some responses were ambiguous or would benefit from clarification. This should be a focus for future in-depth qualitative enquiry.

### Future directions

Several key strands of activity within the UK research community will flow from the work reported here, using the roadmap as a strategic guide to address the needs of under-served groups in research. Through its next phase, the INCLUDE project will focus on the four workstreams shown above. The project will engage with funders and regulators to ensure that the issue of representation of under-served groups is taken into account in funding decisions, regulatory approvals, policies and making sufficient resources available. In addition, the project will partner with others already working in this field, signposting research and delivery teams to existing resources [16], collaborating with others to develop infrastructure and training solutions tailored to the needs of distinct under-served groups, and developing the tools that research teams require to engage with under-served groups and design clinical studies that effectively recruit and retain people (which current studies fail to achieve). Although our focus remains on improving UK research delivery, the generic nature of the barriers we identified means that our roadmap is equally applicable to international initiatives to improve research delivery, including initiatives in lower and middle-income countries. We hope that the INCLUDE framework will provide a structure and a catalyst to improve study design and delivery, improve intervention design and get more people from under-served groups into clinical research, with a consequent improvement in the quality, credibility and applicability of research data and hence better healthcare delivery for a wide range of currently under-served groups.

## Supplementary information

**Additional file 1.** Sample search strategy for targeted scoping review – Medline database search.

**Additional file 2.** Content of survey questions for stakeholder groups.

**Additional file 3.** Description of included studies from targeted scoping review.

## Data Availability

The datasets used and/or analysed during the current study are available from the corresponding author on reasonable request.

## Abbreviations

CINAHL: Cumulative Index to Nursing and Allied Health Literature
CPPR: Community Partnered Participatory Research
DoPHER: Cochrane Database of Systematic Reviews, Database of Promoting Health Effectiveness Reviews
NIHR: National Institute for Health Research
TRoPHI: Trial Register of Promoting Health Interventions
VOICE: Valuing Our Intellectual Capital and Experience
