# Supplementary material for: Developing a roadmap to improve trial delivery for under-served groups: results from a UK multi-stakeholder process
Source: Trials. 2020 Aug 1;21:694. doi: 10.1186/s13063-020-04613-7 (PMC7395975; doi:10.1186/s13063-020-04613-7)
Supplement: Supplementary file 1 — Additional file 1. Sample search strategy for targeted scoping review – Medline database search. [file 13063_2020_4613_MOESM1_ESM.docx]

**Additional file 1: Sample search strategy for targeted scoping review – Medline database search**

| 1 | underrepresent*.mp | (5134) |
| --- | --- | --- |
| 2 | Clinical Trials as topic/ | (183071) |
| 3 | 1 and 2 | (226) |
| 4 | (meta analy* or metanaly* or metaanaly*).ti,ab. | (101240) |
| 5 | ((systematic* or evidence* or literature) adj3 (review* or overview*)).ti,ab. | (312897) |
| 6 | (reference list* or bibliograph* or hand search* or manual search* or relevant journals).ab. | (31537) |
| 7 | (search strategy or search criteria or systematic search or study selection or data extraction or literature review or narrative review).ab. | (73409) |
| 8 | (search* adj4 literature).ab. | (40210) |
| 9 | 3 and (4 or 5 or 6 or 7 or 8) | (30) |
| 10 | Limit 9 to yr=”2013-2018” | (11) |
