# Supplementary material for: Developing a roadmap to improve trial delivery for under-served groups: results from a UK multi-stakeholder process
Source: Trials. 2020 Aug 1;21:694. doi: 10.1186/s13063-020-04613-7 (PMC7395975; doi:10.1186/s13063-020-04613-7)
Supplement: Supplementary file 2 — Additional file 2. Content of survey questions for stakeholder groups. [file 13063_2020_4613_MOESM2_ESM.docx]

**Additional File 2: Content of survey questions for stakeholder groups**

| *Professional survey questions* |  | *Participant survey questions* |
| --- | --- | --- |
|  | | |
| Within the literature, there is a consensus that the meaning of under-representation within clinical trials is context specific and therefore cannot be universally defined. Do you agree with this? Yes, No, Don’t Know. If no, could you please provide us with your definition of under-representation in clinical trials. |  | We have found that it is difficult to be clear about what “under-representation” in clinical trials means. Do you agree with this? Yes - It is difficult to be clear about what “under-representation” means; No - I have a clear understanding of what “under-representation” means and my definition is: |
| Within the published literature there is a general consensus that the following participant groups are often under-represented within clinical trials (full list on forthcoming slide). Are there any groups missing from this list? Yes, No, Don’t Know. If yes, please tell us which group(s) (maximum of three options) and why. |  | It has been suggested that these groups are under-represented in clinical trials (full list on forthcoming slide). Do you think there are any groups that are missing from this list that should be added? Yes, No, Don’t Know. If YES, which group or groups would you add and why? |
| Are there any group(s) that should be removed from this list? Yes, No, Don’t Know. If yes, which group(s)? For each group selected please explain why. |  | Do you think there are any groups on this list that shouldn’t be there? Yes, No, Don’t Know. If YES, which group or groups? For each group selected, please explain why. |
| Across the literature, concerning under-representation within clinical trials, there have been some commonly identified barriers to inclusion (full list on forthcoming slide). Do you think there are any barriers not mentioned here that should be added? Yes, No, Don’t Know. If yes, please tell us which (maximum of five options) and why. |  | Researchers think that there are barriers (obstacles) that stop people from taking part in trials. Some of these could be (full list on forthcoming slide). Do you think there are any barriers that should be added? Yes, No, Don’t Know. If yes, please tell us which (maximum of five options) and why. |
| Are there any barriers on this list that should be removed? Yes, No, Don’t Know If yes, which barrier(s)? For each one selected please explain why. |  | Do you think there are any barriers on this list that shouldn’t be there? Yes, No, Don’t Know. If yes, which barrier(s)? For each group selected please explain why. |
| Within the literature, there are varying innovations in clinical trial delivery and design that are aimed at improving clinical trial participation from under-represented groups. We would like to know about your experiences in attempting to improve under-representation in any of the clinical trials that you have been a part of. Please share any experiences of attempts to improve under-representation that worked. |  | Have you or someone you know ever decided not to take part in a clinical trial when you were asked? Yes, No. If YES, could you tell us why? |
| Please share any experiences of attempts to improve under-representation that failed. |  | Have you or someone you know ever dropped out of a clinical trial before it finished? Yes, No. If YES, could you tell us why? |
| Do you have any examples of innovations designed to improve participation that you would have liked to do but couldn’t (e.g. for ethical, governance, financial, timing reasons)? |  | Have you or someone you know ever been in a clinical trial that was difficult to take part in? Yes, No. If YES, please explain what made taking part difficult. |
| Is there anything else about under-representation in clinical trials not discussed in this survey you would like to raise? |  | Have you or someone you know ever been in a clinical trial that was easy to take part in? Yes, No. If YES, please explain what made taking part easier |
|  |  | Is there anything else that you wish to say about under-representation in clinical trials that hasn’t already been discussed in this survey? |
