# Supplementary material for: Developing a roadmap to improve trial delivery for under-served groups: results from a UK multi-stakeholder process
Source: Trials. 2020 Aug 1;21:694. doi: 10.1186/s13063-020-04613-7 (PMC7395975; doi:10.1186/s13063-020-04613-7)
Supplement: Supplementary file 3 — Additional file 3. Description of included studies from targeted scoping review. [file 13063_2020_4613_MOESM3_ESM.docx]

**Additional File 3. Description of included studies from targeted scoping review**

**Cancer studies**

| **Underserved population(s)** | | **Country** | **Barriers (extracted from Title, Abstract)** | **References** |
| --- | --- | --- | --- | --- |
| Paediatrics | - | USA | Socio-demographic characteristics - age, sex, race/ethnicity, parental language, cancer type, and insurance status | Aristizabal P; Singer J; Cooper R et al. (2015) |
| Children (incl. racial and ethnic minorities) | Lymphohematopoietic (LH) tumours and solid tumours | USA | Focused accrual | Nooka AK; Behera M; Lonial S et al. (2016) |
| Adolescents and young adults (AYAs, 15 to 39 years) | Sarcomas | USA | Cross-network collaboration for cancers that affect AYAs | Davis LE; Janeway KA; Weiss AR et al. (2017) |
| Older patients (defined as age >= 65 years and >= 70 years) | Breast | USA | Disease characteristics, therapy cessation | Freedman RA; Foster JC; Seisler DK et al. (2017) |
| Older adults | - | USA | Trials designed specifically for older adults are rare | Hurria A; Levit LA; Dale W et al. (2015) |
| Older adults | Cervical | Global | Treatment complications and optimisation of outcomes | Venkatesulu BP; Mallick S; Rath GK (2017) |
| Older adults | Non-small-cell lung cancer (NSCLC) | USA | Multiple chronic conditions, polypharmacy, geriatric syndromes, and heterogeneity | Presley CJ; Reynolds CH; Langer CJ (2017) |
| Older adults | Lung | USA | Medical comorbidities | Schulkes KJ; Nguyen C; van den Bos F; et al. (2016) |
| Older adults | Pancreatic | Global | Medical comorbidities, functional status | Garcia G; Odaimi M (2017) |
| Older adults | Prostate | Global | Medical comorbidities, functional status, clinical management | Caffo O; Maines F; Rizzo M et al. (2016) |
| Older adults | - | USA | Balance the risks and benefits of existing cancer therapies; available clinical trial end points pertinent to the older adult population (preservation of function, cognition, and independence) | Hurria A; Dale W; Mooney M et al. (2014) |
| Women | Gynaecological malignancies | USA | Lack of institutional funding for ‘orphan’ cancers | Hall, JB (2015) |
| Women (incl. Black, Hispanic, and elderly) | Gynaecological malignancies | USA | Overrepresentation of privately insured women; National Cancer Institute (NCI) funded trials vs incident population | Mishkin G; Minasian LM; Kohn EC et al. (2016) |
| Women and minorities | - | Global | Defining, recruiting, and reporting | Kwiatkowski K; Coe K; Bailar JC et al. (2013) |
| Minorities | - | USA | Topic placement and topic framing (communication) | Barton E; Eggly S; Winckles A et al. (2014) |
| Minorities, the elderly and persons with more advanced disease | Multiple myeloma (MM) | USA | Industry-sponsored trials vs National Cancer Institute (NCI) or investigator-sponsored trials | Costa LJ; Hari PN; Kumar SK (2016) |
| Minority groups (race, socioeconomic status) | - | USA | Increased cancer clinical trial complexity, financial burden to institution | Gerber DE; Lakoduk AM; Priddy LL et al. (2015) |
| Ethnically diverse cancer populations | - | USA and Canada | Culturally and linguistically appropriate outreach, education, research studies accessible in local communities; infrastructure to support engagement of key stakeholders, clinicians, and organisations serving minority communities; testimonials by ethnically diverse cancer survivors; availability of medical interpretation services | Napoles, A; Cook, E; Ginossar, T et al. (2017) |
| Hispanic cancer populations | - | USA | Spanish-language multimedia | Wells KJ; McIntyre J; Gonzalez LE et al. (2013) |
| African American cancer populations | Lung, breast, prostate or colorectal | USA | Information gathering, intrapersonal perspectives, and interpersonal influences (presence or absence of decision regret and satisfaction) | Wenzel JA; Mbah O; Xu J; Moscou-Jackson G et al. (2015) |
| African Americans | - | USA | Differences in the way clinical trials are discussed in oncology visits with African American vs. White patients | Eggly S; Barton E; Winckles A et al. (2015) |
| African Americans | - | USA | Negative attitudes towards clinical trials, low levels of knowledge and awareness regarding CCTs, religious beliefs, and structural barriers such as transportation, childcare, and access to health care | Rivers D; August EM; Sehovic I et al. (2013) |
| African American men | Prostate | USA | Socioeconomic status, education and awareness, eligibility barriers (comorbidities), willingness to participate, cultural barriers, type of institution where patients are treated | Ahaghotu C; Tyler R; Sartor O (2016) |
| American Indians and Alaska Natives (AIs/ANs) – University students | - | USA | Unknown attitudes toward clinical trial participation | Sprague D; Russo J; LaVallie DL et al. (2013) |
| Socioeconomic status | - | Global | Socioeconomic deprivation | Sharrocks K; Spicer J; Camidge DR et al. (2014) |
| Cancer trial recruitment processes | - | USA | Low research literacy; few tools exist for facilitating dialogue between researchers and potential research participants during the recruitment process | Torres S; de la Riva EE; Tom LS et al. (2015) |

**Non-cancer**

| **Underrepresented population(s)** | | **Country** | **Barriers (extracted from Title, Abstract)** | **References** |
| --- | --- | --- | --- | --- |
| Paediatrics | Sickle cell disease (SCD) | USA | Decision-making processes of youth with SCD and their caregivers regarding enrolment in clinical trial research incl. potential harm, potential benefits, manageable study demands, trust in medical staff | Patterson CA; Chavez V; Mondestin V et al. (2015) |
| Older adults, patients aged >=75 | Cardiovascular disorders | USA | Medical comorbidities, significant physical or cognitive disabilities, frailty, or residence in a nursing home or assisted living facility | Rich MW; Chyun DA; Skolnick AH et al. (2016) |
| Older adults (aged >=75) with multiple comorbidities, concomitant treatments and/or frailty | Aging populations of Europe | Europe | Recruitment, process of informed consent, role of ethics committees | Diener, L; Hugonot-Diener, L; Alvino, S et al. (2013) |
| Older adults | Multimorbidity | Global | Trial methodology, disease-centred indicators vs patient-centred indicators, education of future geriatricians, lack of public awareness campaigns | Marengoni A (2013) |
| Older adults | Transplant recipients | USA | Clinician, sponsor, and investigator engagement with the FDA | Meyer JM; Archdeacon P; Albrecht R et al. (2013) |
| Older adults | Interventional trials | Global | Studies are few, small in scope (even for conditions that affect almost exclusively the elderly) | Bourgeois FT; Olson KL; Tse T et al. (2016) |
| Men, older adults | Osteoporosis | Global | Trials of interventions that are not required to be registered in ClinicalTrials.gov may be underrepresented; majority of sampled trials enrolled only women with research trials also excluding older adults (>65 years of age, >75 years of age) | Barnard K; Lakey WC; Batch BC et al. (2016) |
| Women | Information gap on medical device safety and effectiveness between sexes | USA | Encouraging female participation | Zusterzeel R; O'Callaghan KM; Canos DA et al. (2016) |
| Women (Military) | Retention of military women in research studies | USA | Logistical, cultural, social, ethical, and methodological issues (lengthy deployments, unpredictable military exercises, and foreign assignments) | Braun LA; Kennedy HP; Sadler LS; Dixon J (2015) |
| Women (Pregnancy) | Socioecological model | Global | Influence and reach of prenatal providers, engagement with existing community-based organisations | Frew PM; Saint-Victor DS; Isaacs MB et al. (2014) |
| African American women | Community-Engaged Research Core (CERC) | USA | Participant retention, drug adherence | Johnson DA; Joosten YA; Wilkins CH et al. (2015) |
| African Americans, Latinos | Community Partnered Participatory Research (CPPR) | USA | Communities are often described as difficult to engage in research | Sankare IC; Bross R; Brown AF et al. (2015) |
| African Americans, Latinos | Solutions from African Americans and Latinos reflecting their cultural backgrounds and historical experiences | USA | Costs, recruiting in community contexts, conducting community and individualised patient education, and sharing patient safety information | Ford ME; Siminoff LA; Pickelsimer E et al. (2013) |
| African Americans | Culturally tailored intervention (church/university partnership) | USA | Matching volunteers to appropriate studies | Langford AT; Resnicow K; Beasley DD (2015) |
| African Americans | Obesity | USA | Culturally tailored intervention materials, reporting of outcomes by race | Goode RW; Styn MA; Mendez DD; Gary-Webb TL (2017) |
| African Americans | Culturally Competent Strategies for Recruitment and Retention | USA | Distrust, compensation, education disadvantage, lack of interest, and inability to have study partner | Otado J; Kwagyan J; Edwards D et al. (2015) |
| African Americans | Church-based educational intervention | USA | Intention to obtain clinical trial information and intention to join a clinical trial | Frew PM; Schamel JT; O'Connell KA et al. (2015) |
| Asian Americans | Addressing community concerns | USA | More information required about clinical trials and benefits to science and the larger community; role of health-care providers in recruitment; basing recruitment goals on percentage representation in most US geographic areas does not provide sufficient numbers to allow for analysis of minorities | Ma GX; Seals B; Tan Y; Wang SY et al. (2014) |
| Minorities | HIV/AIDS research | USA | Enrolment strategies | Castillo-Mancilla JR; Cohn SE; Krishnan S et al. (2014) |
| Ethnic minorities | Cardiovascular disease | USA | Reporting of ethnicity/race | Zhang T; Tsang W; Wijeysundera HC et al. (2013) |
| Ethnic minorities | Community-engaged interventions | USA | Clinical trial awareness, opportunity to participate, and acceptance of enrolment | Heller C; Balls-Berry JE; Nery JD et al. (2014) |
| Ethnic minorities | Cystic fibrosis (CF) | Global | Failure to report the racial or ethnic background of study subjects | McGarry ME; McColley SA (2016) |
| Ethnic minorities | Mental health (bipolar disorder, schizophrenia, and major depression, along with trials of children and adults with attention-deficit hyperactivity disorder) | USA | Few ethnic-specific analyses are being conducted as they relate to racial-ethnic minority groups in the workforce with identified mental health disorders | Santiago CD; Miranda J (2014) |
| Persons Who Use Drugs (PWUD) | HIV-related research | USA | Individual (participant), institutional, and recruiter-level challenges | Batista P; Deren S; Banfield A et al. (2016) |
| Cognitive impairment | Delirium | Global | Influence of dementia | de Jonghe, A; van de Glind, EM; van Munster, BC et al. (2014) |
| Alzheimer’s Disease | Psychosocial interventions/gender bias | Global | Clinical trials do not currently routinely control for gender bias | Baron, S; Ulstein, I; Werheid, K. (2015) |
| Low-income countries | Clinical trials in Africa (Ethiopia) | Ethiopia | Cross-country collaborations, international funding support, motivation of academic staff to conduct clinical trials and the commitment and engagement of the leadership in research, administrative capacity, research infrastructure as well as financial support, enhanced university-industry linkage and translation of research findings into locally relevant evidence | Fekadu A; Teferra S; Hailu A et al. (2014) |
| Low income and minority individuals | Obesity | Global | Well-controlled behavioural interventions, access to treatment is often limited | Harvey JR; Ogden DE (2014) |
| Women, children, older adults, common comorbidities | Generalizability | Global | Gender, health insurance, comorbidities, age & motivation | Wang W; Ma Y; Chen H (2017) |
| Children, older adults, pregnant and lactating women, and individuals with physical and intellectual disabilities | Improving public health | USA | Paediatric drug doses were based on extrapolation from adults, comorbidities, pharmacodynamics/pharmacokinetic study designs, ability to provide informed consent, identification of subpopulations | Spong, CY; Bianchi, DW (2018) |
| Older adults, socially deprived, those with multiple comorbidities | Incentive payment to improve recruitment to clinical trials | United Kingdom | Socially deprived geographic areas | Jennings CG; MacDonald TM; Wei L et al. (2015) |
| Women, older adults, racial and ethnic minorities | Pivotal trials supporting 2011-2013 U.S. Food and Drug Administration approvals | Global | Demography | Downing NS; Shah ND; Neiman JH et al. (2016) |

**References for literature review:**

Ahaghotu C., Tyler R., and Sartor O. (2016) ‘African American Participation in Oncology Clinical Trials--Focus on Prostate Cancer: Implications, Barriers, and Potential Solutions’, *Clinical Genitourinary Cancer*, 14(2), pp. 105-16. doi: 10.1016/j.clgc.2015.12.003.

Aristizabal P., Singer J., Cooper R., et al. (2015) ‘Participation in pediatric oncology research protocols: Racial/ethnic, language and age-based disparities’, *Pediatric Blood & Cancer*, 62(8), pp. 1337-44. doi: 10.1002/pbc.25472.

Barnard K., Lakey W.C., Batch B.C., Chiswell K., Tasneem A., and Green J.B. (2016) ‘Recent Clinical Trials in Osteoporosis: A Firm Foundation or Falling Short?’, *PLoS ONE*, 11(5), e0156068. doi: 10.1371/journal.pone.015606.

Baron S., Ulstein I., and Werheid K. (2015) ‘Psychosocial interventions in Alzheimer's disease and amnestic mild cognitive impairment: Evidence for gender bias in clinical trials’*, Aging & Mental Health*, 19(4), pp. 290-305. doi: 10.1080/13607863.2014.938601.

Barton E., Eggly S., Winckles A., and Albrecht T.L. (2014) ‘Strategies of persuasion in offers to participate in cancer clinical trials I: Topic placement and topic framing’, *Journal of Community Medicine*, 11(1), pp. 1-14.

Batista P., Deren S., Banfield A., et al. (2016) ‘Challenges in Recruiting People Who Use Drugs for HIV-Related Biomedical Research: Perspectives from the Field’, *AIDS Patient Care and STDS*, 30(8), pp. 379-84. doi: 10.1089/apc.2016.0135.

Bourgeois F.T., Olson K.L., Tse T., Ioannidis J.P., and Mandl K.D. (2016) ‘Prevalence and Characteristics of Interventional Trials Conducted Exclusively in Elderly Persons: A Cross-Sectional Analysis of Registered Clinical Trials’, *PLoS ONE*, 11(5), e0155948. doi: 10.1371/journal.pone.0155948.

Braun L.A., Kennedy H.P., Sadler L.S., and Dixon J. (2015) ‘Research on U.S. Military Women: Recruitment and Retention Challenges and Strategies’, *Military Medicine*, 180(12), pp. 1247-55. doi: 10.7205/MILMED-D-14-00601.

Braun L.A., Kennedy H.P., Sadler L.S., and Dixon J. (2015) ‘Research on U.S. Military Women: Recruitment and Retention Challenges and Strategies’, *Military Medicine*, 180(12), pp. 1247-55. doi: 10.7205/MILMED-D-14-00601.

Caffo O., Maines F., Rizzo M., Kinspergher S., and Veccia A. (2016) ‘Metastatic castration-resistant prostate cancer in very elderly patients: challenges and solutions’, *Clinical Interventions in Aging*, 12, pp. 19-28. doi: 10.2147/CIA.S98143.

Castillo-Mancilla J.R., Cohn S.E., Krishnan S., et al. (2014) ‘Minorities remain underrepresented in HIV/AIDS research despite access to clinical trials’, *HIV Clinical Trials*, 15(1), pp. 14-26. doi: 10.1310/hct1501-14.

Costa L.J., Hari P.N., and Kumar S.K. (2016) ‘Differences between unselected patients and participants in multiple myeloma clinical trials in US: a threat to external validity’, *Leukemia & Lymphoma*, 57(12), pp. 2827-2832. ETI Brief – Workstream 1 (Final Draft) | Page 17 of 21

Davis L.E., Janeway K.A., Weiss A.R., et al. (2017) ‘Clinical trial enrollment of adolescents and young adults with sarcoma’, Cancer, 123(18), pp. 3434-3440. doi: 10.1002/cncr.30757.

de Jonghe, A., van de Glind E.M., van Munster B.C., and de Rooij, S.E. (2014) ‘Underrepresentation of patients with pre-existing cognitive impairment in pharmaceutical trials on prophylactic or therapeutic treatments for delirium: A systematic review’, Journal of Psychosomatic Research, 76(3), pp. 193-199. doi: 10.1016/j.jpsychores.2013.12.007.

Diener L., Hugonot-Diener L., Alvino S., et al. (2013) ‘Guidance synthesis. Medical research for and with older people in Europe: proposed ethical guidance for good clinical practice: ethical considerations’, The Journal of Nutrition Health and Aging, 17(7), pp. 625-7. doi: 10.1007/s12603-013-0340-0.

Downing N.S., Shah N.D., Neiman J.H., Aminawung J.A., Krumholz H.M., and Ross J.S. (2016) ‘Participation of the elderly, women, and minorities in pivotal trials supporting 2011-2013 U.S. Food and Drug Administration approvals’, Trials, 17, pp. 199. doi: 10.1186/s13063-016-1322-4.

Eggly S., Barton E., Winckles A., Penner L.A., and Albrecht T.L. (2015) ‘A disparity of words: racial differences in oncologist-patient communication about clinical trials’, Health Expectations, 18(5), pp. 1316-26. doi: 10.1111/hex.12108.

Fekadu A., Teferra S., Hailu A., et al. (2014) ‘International Clinical Trial Day and clinical trials in Ethiopia and Africa’, Trials, 15, pp. 493. doi: 10.1186/1745-6215-15-493.

Ford M.E., Siminoff L.A., Pickelsimer E., et al. (2013) ‘Unequal burden of disease, unequal participation in clinical trials: solutions from African American and Latino community members’, Health Social Work, 38(1), pp. 29-38.

Freedman R.A., Foster J.C., Seisler D.K., et al. (2017) ‘Accrual of Older Patients With Breast Cancer to Alliance Systemic Therapy Trials Over Time: Protocol A151527’, Journal of Clinical Oncology, 35(4), pp. 421-431. doi: 10.1200/JCO.2016.69.4182.

Frew P.M., Saint-Victor D.S., Isaacs M.B., et al. (2014) ‘Recruitment and retention of pregnant women into clinical research trials: an overview of challenges, facilitators, and best practices’, Clinical Infectious Diseases, 59 Suppl 7, S400-7. doi: 10.1093/cid/ciu726.

Frew P.M., Schamel J.T., O'Connell K.A., Randall L.A., and Boggavarapu S. (2015) ‘Results of a Community Randomized Study of a Faith-Based Education Program to Improve Clinical Trial Participation among African Americans’, International Journal of Environmental Research and Public Health, 13(1):ijerph13010041. doi: 10.3390/ijerph13010041.

Garcia, G. and Odaimi, M. (2017) ‘Systemic Combination Chemotherapy in Elderly Pancreatic Cancer: a Review’, Journal of Gastrointestinal Cancer, 48(2), pp. 121-128. doi: 10.1007/s12029-017-9930-0.

Gerber D.E., Lakoduk A.M., Priddy L.L., Yan J., and Xie X.J. (2015) ‘Temporal Trends and Predictors for Cancer Clinical Trial Availability for Medically Underserved Populations’, Oncologist, 20(6), pp. 674-82. doi: 10.1634/theoncologist.2015-008. ETI Brief – Workstream 1 (Final Draft) | Page 18 of 21

Gerber D.E., Lakoduk A.M., Priddy L.L., Yan J., and Xie X.J. (2015) ‘Temporal Trends and Predictors for Cancer Clinical Trial Availability for Medically Underserved Populations’, *Oncologist*, 20(6), pp. 674-82. doi: 10.1634/theoncologist.2015-008.

Goode R.W., Styn M.A., Mendez D.D., and Gary-Webb T.L. (2017) ‘African Americans in Standard Behavioral Treatment for Obesity, 2001-2015: What Have We Learned?’, Western Journal of Nursing Research, 39(8), pp. 1045-1069. doi: 10.1177/0193945917692115.

Hall J.B. (2015) ‘Underrepresentation of women in clinical trials: why gynecologic oncologists are worried’, Obstetrics & Gynecology, 125(6), pp. 1500-1. doi: 10.1097/AOG.0000000000000895.

Harvey J.R., and Ogden D.E. (2014) ‘Obesity treatment in disadvantaged population groups: where do we stand and what can we do?’, Preventative Medicine, 68, pp. 71-5. doi: 10.1016/j.ypmed.2014.05.015.

Heller C., Balls-Berry J.E., Nery J.D., et al. (2014) ‘Strategies addressing barriers to clinical trial enrollment of underrepresented populations: a systematic review’, Contemporary Clinical Trials, 39(2), pp. 169-82. doi: 10.1016/j.cct.2014.08.004.

Hurria A., Dale W., Mooney M., et al. (2014) ‘Designing therapeutic clinical trials for older and frail adults with cancer: U13 conference recommendations’, Journal of Clinical Oncology, 32(24), pp. 2587-94.

Hurria A., Levit L.A., Dale W., et al. (2015) ‘Improving the Evidence Base for Treating Older Adults With Cancer: American Society of Clinical Oncology Statement’, Journal of Clinical Oncology, 33(32), pp. 3826-33. doi: 10.1200/JCO.2015.63.0319.

Jennings C.G., MacDonald T.M., Wei L., Brown M.J., McConnachie L., and Mackenzie I.S. (2015) ‘Does offering an incentive payment improve recruitment to clinical trials and increase the proportion of socially deprived and elderly participants?’, Trials, 16, pp. 80. doi: 10.1186/s13063-015-0582-8.

Johnson D.A., Joosten Y.A., Wilkins C.H., and Shibao C.A. (2015) ‘Case Study: Community Engagement and Clinical Trial Success: Outreach to African American Women’, Clinical Translational Science, 8(4), pp. 388-90. doi: 10.1111/cts.12264.

Kwiatkowski K., Coe K., Bailar J.C., and Swanson G.M. (2013) ‘Inclusion of minorities and women in cancer clinical trials, a decade later: Have we improved?’, Cancer, 119(16), pp. 2956-63. doi: 10.1002/cncr.28168.

Langford A.T., Resnicow K., and Beasley D.D. (2015) ‘Outcomes from the Body & Soul Clinical Trials Project: a university-church partnership to improve African American enrollment in a clinical trial registry’, Patient Education and Counseling, 98(2), pp. 245-50. doi: 10.1016/j.pec.2014.10.018.

Ma G.X., Seals B., Tan Y., Wang S.Y., Lee R., and Fang C.Y. (2014) ‘Increasing Asian American participation in clinical trials by addressing community concerns’, Clinical Trials, 11(3), pp. 328-335. doi: 10.1177/1740774514522561.

Marengoni A. (2013) ‘Guidelines for elderly patients with multimorbidity: how to cope with a dark night without fear’, Aging Clinical and Experimental Research, 25(6), pp. 703-5. doi: 10.1007/s40520-013-0072-3. ETI Brief – Workstream 1 (Final Draft) | Page 19 of 21

McGarry M.E. and McColley S.A. (2016) ‘Minorities Are Underrepresented in Clinical Trials of Pharmaceutical Agents for Cystic Fibrosis’, Annals of the American Thoracic Society, 13(10), pp. 1721-1725.

Meyer J.M., Archdeacon P., and Albrecht R. (2013) ‘FDA perspective: enrolment of elderly transplant recipients in clinical trials’, Transplantation, 95(7), pp. 916-8. doi: 10.1097/TP.0b013e31828279d9.

Mishkin G., Minasian L.M., Kohn E.C., Noone A.M., and Temkin S.M. (2016) ‘The generalizability of NCI-sponsored clinical trials accrual among women with gynecologic malignancies’, Gynecologic Oncology, 143(3), pp. 611-616. doi: 10.1016/j.ygyno.2016.09.026.

Napoles A., Cook E., Ginossar T., Knight K.D., and Ford M.E. (2017) ‘Applying a Conceptual Framework to Maximize the Participation of Diverse Populations in Cancer Clinical Trials’, Advanced Cancer Research, 133, pp. 77-94. doi: 10.1016/bs.acr.2016.08.004.

Nooka A.K., Behera M., Lonial S., Dixon M.D., Ramalingam S.S., and Pentz R.D. (2016) ‘Access to Children's Oncology Group and Pediatric Brain Tumor Consortium phase 1 clinical trials: Racial/ethnic dissimilarities in participation’, Cancer, 122(20), pp. 3207-3214. doi:10.1002/cncr.30090.

Otado J., Kwagyan J., Edwards D., Ukaegbu A., Rockcliffe F., and Osafo N. (2015) ‘Culturally Competent Strategies for Recruitment and Retention of African American Populations into Clinical Trials’, Clinical Translational Science, 8(5), pp. 460-6. doi: 10.1111/cts.12285.

Patterson C.A., Chavez V., Mondestin V., Deatrick J., Li Y., and Barakat L.P. (2015) ‘Clinical Trial Decision Making in Pediatric Sickle Cell Disease: A Qualitative Study of Perceived Benefits and Barriers to Participation’, Journal of Pediatric Hematology/Oncology, 37(6), pp. 415-22. doi: 10.1097/MPH.0000000000000216.

Presley C.J., Reynolds C.H., and Langer C.J. (2017) ‘Caring for the Older Population With Advanced Lung Cancer’, American Society of Clinical Oncology, 37, pp. 587-596. doi: 10.14694/EDBK_179850.

Rich M.W., Chyun D.A., Skolnick A.H., et al. (2016) ‘Knowledge Gaps in Cardiovascular Care of the Older Adult Population: A Scientific Statement From the American Heart Association, American College of Cardiology, and American Geriatrics Society’, Journal of the American College of Cardiology, 67(20), pp. 2419-2440. doi: 10.1016/j.jacc.2016.03.004.

Rivers D., August E.M., Sehovic I., Lee Green B., and Quinn G.P. (2013) ‘A systematic review of the factors influencing African Americans' participation in cancer clinical trials’, Contemporary Clinical Trials, 35(2), pp. 13-32. doi :10.1016/j.cct.2013.03.007.

Sankare I.C., Bross R., Brown A.F., et al. (2015) ‘Strategies to Build Trust and Recruit African American and Latino Community Residents for Health Research: A Cohort Study’, Clinical and Translational Science, 8(5), pp. 412-20. doi: 10.1111/cts.12273. ETI Brief – Workstream 1 (Final Draft) | Page 20 of 21

Santiago C.D., and Miranda J. (2014) ‘Progress in improving mental health services for racial-ethnic minority groups: a ten-year perspective’, Psychiatric Services, 65(2), pp. 180-5. doi: 10.1176/appi.ps.201200517.

Sharrocks K., Spicer J., Camidge D.R., and Papa S. (2014) ‘The impact of socioeconomic status on access to cancer clinical trials’, British Journal of Cancer, 111(9), pp. 1684-7. doi: 10.1038/bjc.2014.108.

Schulkes K.J., Nguyen C., van den Bos F., van Elden L.J., and Hamaker M.E. (2016) ‘Selection of Patients in Ongoing Clinical Trials on Lung Cancer’, Lung, 194(6), pp. 967-974.

Sprague D., Russo J., LaVallie D.L., and Buchwald D. (2013) ‘Barriers to cancer clinical trial participation among American Indian and Alaska Native tribal college students’, Journal of Rural Health, 29(1), pp. 55-60. doi: 10.1111/j.1748-0361.2012.00432.x.

Spong, C.Y. and Bianchi, D.W. (2018) ‘Improving Public Health Requires Inclusion of Underrepresented Populations in Research’, Journal of the American Medical Association, 319(4), pp. 337-338. doi: 10.1001/jama.2017.19138.

Torres S., de la Riva E.E., Tom L.S., et al. (2015) ‘The Development of a Communication Tool to Facilitate the Cancer Trial Recruitment Process and Increase Research Literacy among Underrepresented Populations’, Journal of Cancer Education, 30(4), pp. 792-8. doi: 10.1007/s13187-015-0818-z.

Venkatesulu B.P., Mallick S., and Rath G.K. (2017) ‘Patterns of care of cervical cancer in the elderly: A qualitative literature review’, Journal of Geriatric Oncology, 8(2), pp. 108-116. doi: 10.1016/j.jgo.2016.12.004.

Wang, W., Ma, Y., Huang, Y. and Chen, H. (2017) ‘Generalizability analysis for clinical trials: a simulation study’, Statistics in Medicine, 36(10), pp. 1523-1531. doi: 10.1002/sim.7238.

Wells K.J., McIntyre J., Gonzalez L.E., et al. (2013) ‘Feasibility trial of a Spanish-language multimedia educational intervention’, Clinical Trials, 10(5), pp. 767-74. doi: 10.1177/1740774513495984.

Wenzel J.A., Mbah O., Xu J., et al. (2015) ‘A Model of Cancer Clinical Trial Decision-making Informed by African-American Cancer Patients’, Journal of Racial and Ethnic Health Disparities, 2(2), pp. 192-9. doi: 10.1007/s40615-014-0063-x.

Zhang T., Tsang W., Wijeysundera H.C., and Ko D.T. (2013) ‘Reporting and representation of ethnic minorities in cardiovascular trials: a systematic review’, American Heart Journal, 166(1), pp. 52-7. doi: 10.1016/j.ahj.2013.03.022.

Zusterzeel R., O'Callaghan K.M., Canos D.A., Sanders W.E., Marinac-Dabic D., and Strauss D.G. (2016) ‘Improving the Safety and Effectiveness of Medical Device Therapy in Women’, Journal of Women’s Health, 25(5), pp. 428-30. doi: 10.1089/jwh.2015.5605.
